# Sonographic Detection of a Lumbar Facet Joint Abscess with Epidural Involvement

**DOI:** 10.1055/a-2972-5876

**Published:** 2026-10-16

**Authors:** Eckehart Schöll, Anja Carolin Mair, Rainer Jürgen Litz

**Affiliations:** 1 Department of Emergency Medicine 30251 Bethesda Spital AGBaselBSSwitzerland; 2 USRA Medical EducationIndependent ResearcherAugsburgGermany

## Introduction

Septic arthritis of the spinal facet joint is an uncommon but likely under-recognized cause of back pain and may account for up to 20% of spontaneous pyogenic spinal infections (J. Narváez et al. *Semin Arthritis Rheum* 2006; 35: 272–283). The lumbar spine is most commonly affected, and diagnosis may be delayed because clinical symptoms initially mimic mechanical back pain. Magnetic resonance imaging (MRI) is considered the imaging modality of choice for detecting facet joint infection and associated epidural or paraspinal abscess formation (K. M. Sin et al. *J Clin Imaging Sci* 2024; 14: 34). However, paraspinal abscesses adjacent to the facet joints may also be detected by ultrasound. We report a case in which ultrasound first revealed a lumbar facet joint abscess with perifocal hyperemia, subsequently confirmed by MRI and surgical findings.

## Case description

A 47-year-old male truck driver presented to the emergency department (ED) with acute left-sided low back pain that had started 5 days earlier after lifting pallets at work. The pain radiated to the posterior left thigh. As “red flags” such as motor or sensory deficits, bladder dysfunction, or fever were initially absent, the clinical presentation was considered uncomplicated low back pain.

The patient was discharged with recommendations for oral analgesic therapy and physiotherapy.

Three days later, he returned because low back pain persisted; however, he had not consistently taken the prescribed analgesics and had not yet started physiotherapy. Neurological examination remained largely unremarkable. After intravenous analgesia with ketorolac and metamizole, the symptoms improved rapidly, and the patient was again discharged with instructions for consistent analgesic therapy and mobilization.

On the following day, the patient presented again with markedly worsening back pain and newly developed fever (38.5°C), as well as tachycardia at rest (110/min). Blood pressure was within the normal range (133/88 mm Hg). Physical examination now revealed mild hypesthesia of the anterolateral left thigh. Laboratory tests showed significantly elevated inflammatory markers (C-reactive protein 224 mg/L and leukocytes 10.6 G/L).

Lumbar ultrasound examination was performed using a curved array transducer (Samsung V8, CA1-7S, musculoskeletal setting). A heteroechoic fluid collection measuring approximately 3.5×3×3 cm adjacent to the left L4/5 facet joint ( Fig 1a , 2a ) was detected, with extension into the paraspinal musculature and toward the psoas region ( Fig. 3 ). Color Doppler ultrasound demonstrated pronounced perifocal hyperemia indicating active inflammation ( Fig. 4 ). Blood cultures were obtained.

**Fig. 1:**
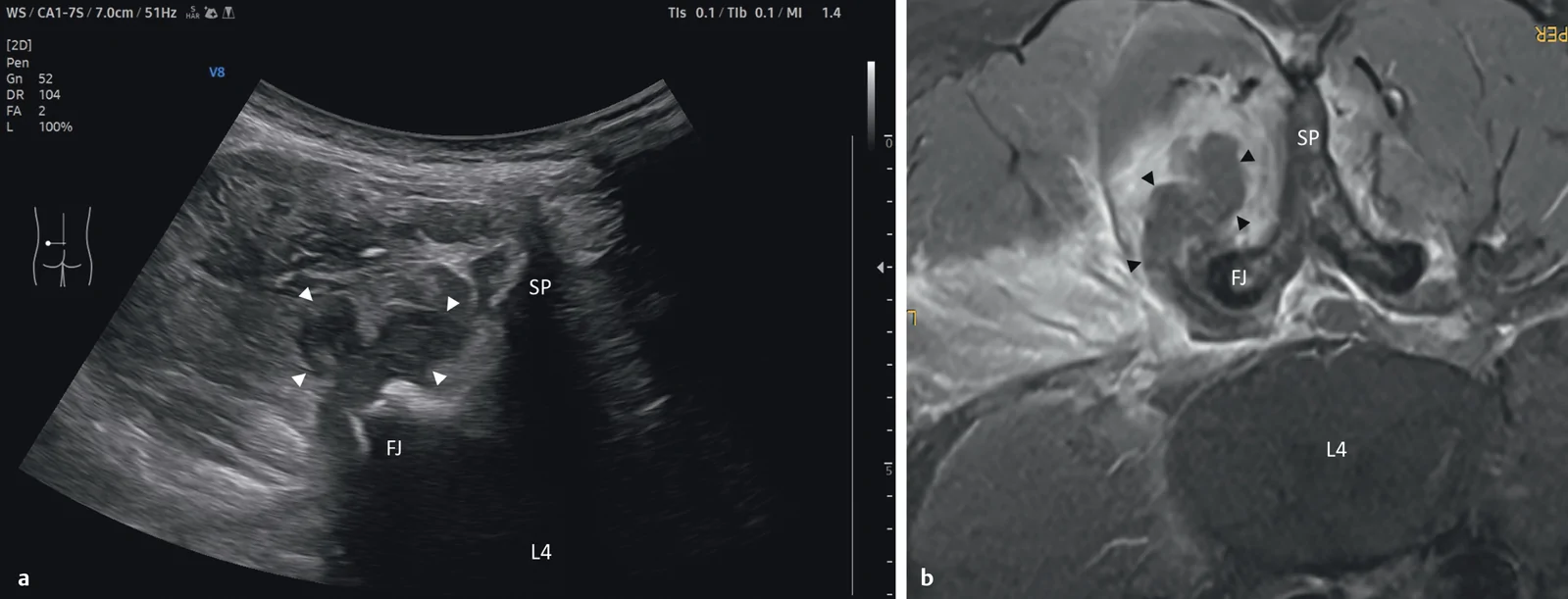
( **a** ) Transverse ultrasound of the lumbar region showing a heteroechoic fluid collection adjacent to the left L4/5 facet joint (white arrowheads). The SP and the level of the L4 vertebra serve as anatomical landmarks. FJ, facet joint; L4, vertebral body L4; SP, spinous process. ( **b** ) Corresponding axial contrast-enhanced MRI demonstrating a paraspinal abscess arising from the left L4/5 facet joint (black arrowheads).

**Fig. 2:**
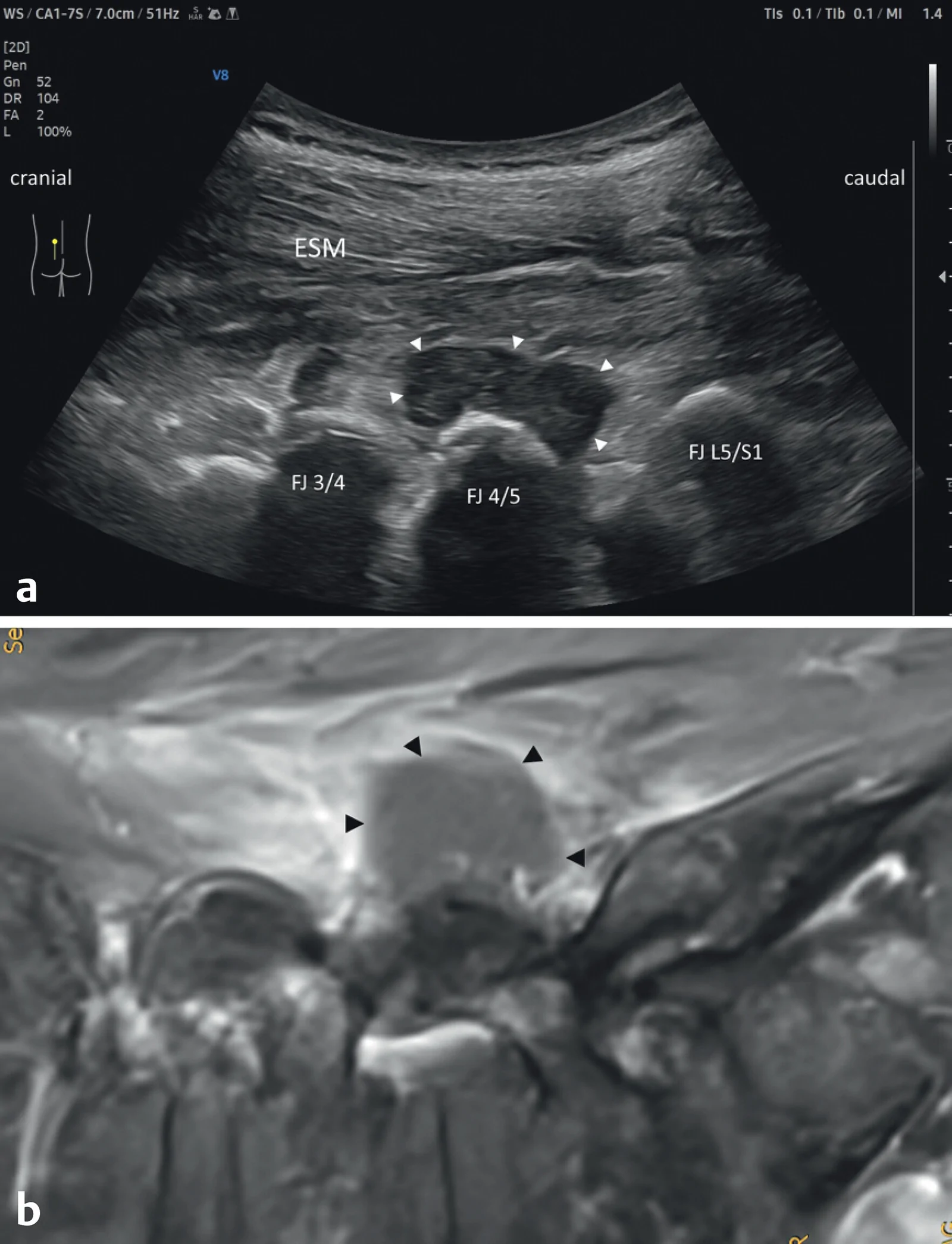
( **a** ) Longitudinal ultrasound of the lumbar region demonstrating the paraspinal fluid collection (white arrowheads) adjacent to the left L4/5 facet joint. ( **b** ) Corresponding sagittal MRI demonstrating the paraspinal abscess (black arrowheads) and inflammatory changes of the posterior spinal elements.

**Fig. 3:**
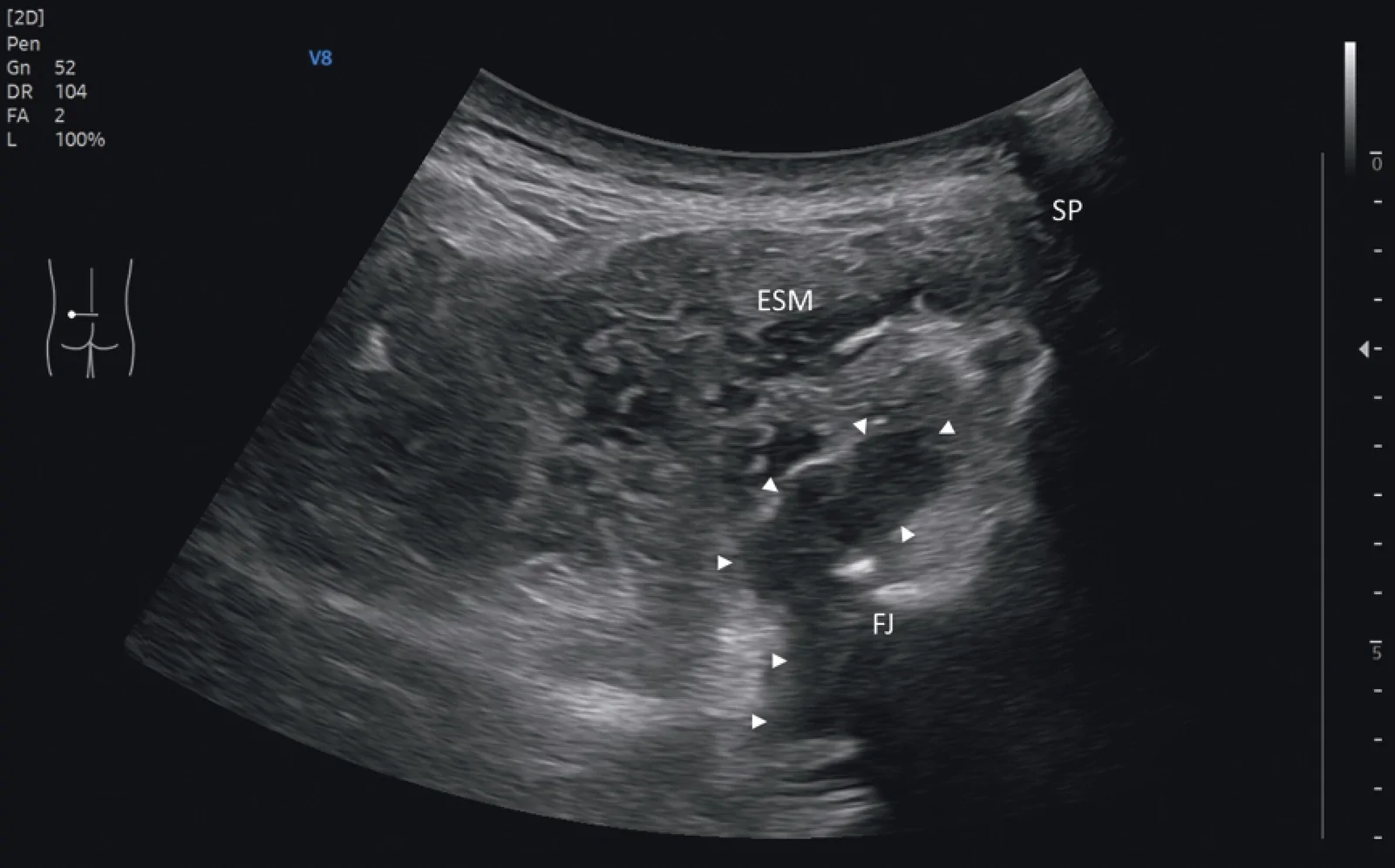
A transverse ultrasound image showing extension of the fluid collection from the left L4/5 facet joint (FJ) into the deeper paraspinal musculature (white arrowheads). ESM, erector spinae muscle; SP, spinous process.

**Fig. 4:**
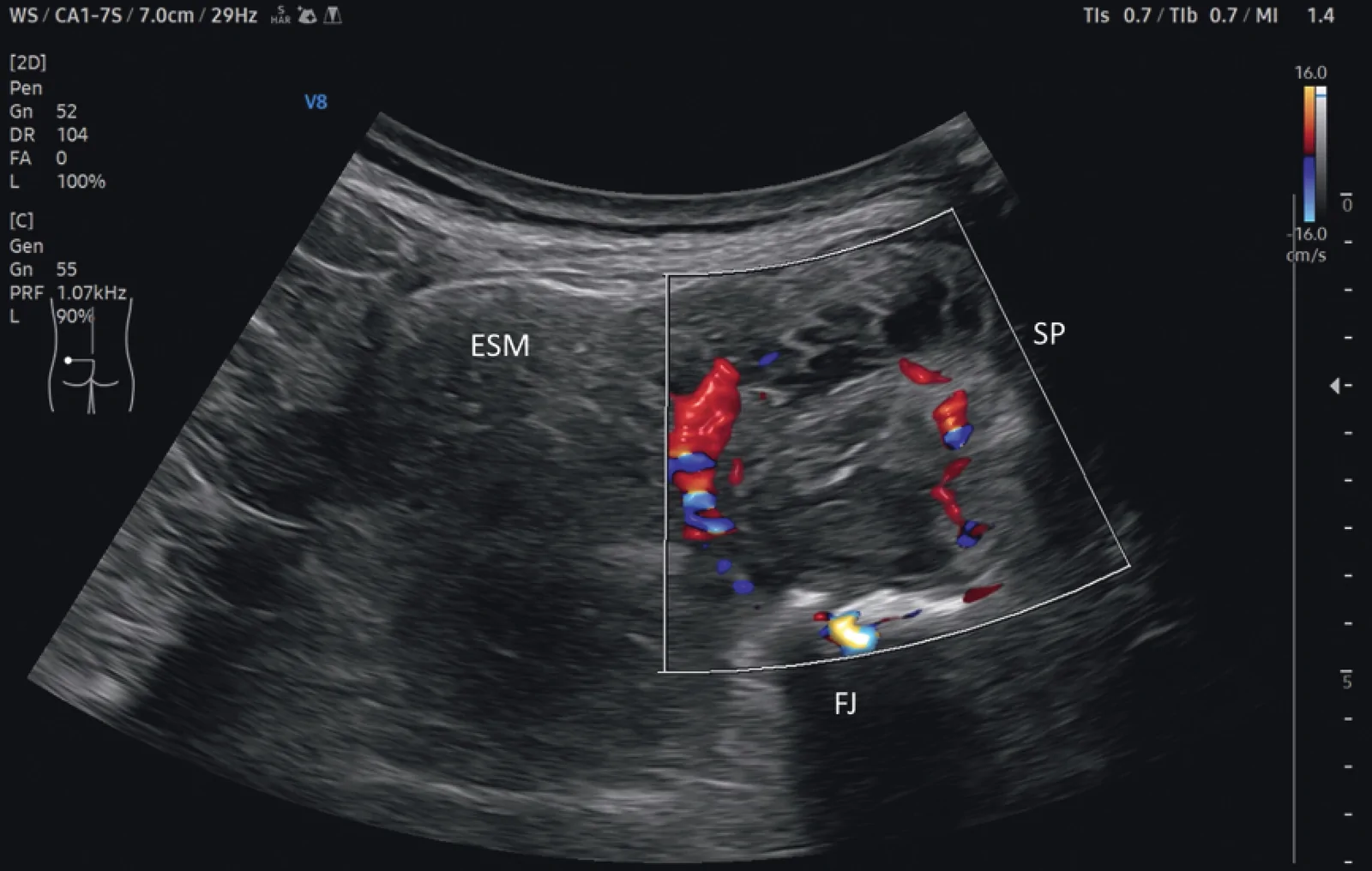
Color Doppler ultrasound demonstrating marked perifocal hyperemia surrounding the paraspinal fluid collection, consistent with active inflammatory changes.

Contrast-enhanced MRI was subsequently performed and confirmed septic arthritis of the left L4/5 facet joint with a paraspinal abscess ( Fig. 1b , 2b ) and epidural involvement with beginning compression of the dural sac.

The patient underwent immediate surgical abscess evacuation and microsurgical left-sided decompression at the L4/5 level. Microbiological and histological analyses revealed *Staphylococcus aureus* in all blood cultures and intraoperative tissue samples. Due to postoperative wound healing complications with persistent secretion, surgical wound revision with debridement and drainage placement was required 5 days later.

Intravenous antibiotic therapy with cefazolin was administered for 4 weeks, followed by oral antibiotic therapy with clindamycin for 2 weeks. The patient recovered completely and was discharged after 14 days. Antibiotic therapy was continued for a total duration of 6 weeks, resulting in complete resolution of the infection.

## Discussion

Septic arthritis of the spinal facet joints is an uncommon spinal infection that predominantly affects the lumbar spine. It accounts for approximately 1% of all septic arthritis cases (J. Ross et al. *Open Forum Infect Dis* 2024). The condition typically presents with nonspecific symptoms such as localized back pain and may initially be misinterpreted as mechanical lumbar pain, which can delay diagnosis.

MRI is considered the imaging modality of choice because it accurately demonstrates facet joint infection and associated epidural or paraspinal extension (K. M. Sin et al. *J Clin Imaging Sci* 2024; 14: 34). Facet joint infections may lead to epidural abscesses or paraspinal soft tissue involvement (M. S. Baltz et al. *Clin Orthop Relat Res* 1997; 339: 109–112; S. Al-Risi et al. *Int J Case Rep Images* 2025; 16: 5–10). Early diagnosis is therefore crucial to prevent neurological complications.

Ultrasound may provide important early diagnostic clues in patients with paraspinal abscess formation. It is widely available in EDs and can be performed rapidly without radiation exposure. This is particularly relevant in emergency settings, where ultrasound may serve as an immediate bedside tool to guide subsequent imaging.

In the present case, lumbar ultrasound revealed a heteroechoic fluid collection adjacent to the L4/5 facet joint with extension into the paraspinal musculature. Color Doppler imaging demonstrated pronounced perifocal hyperemia indicating active inflammation. These findings raised the suspicion of a deep paraspinal infection and prompted immediate MRI. MRI remains mandatory, as it allows the reliable assessment of epidural involvement, which cannot be excluded by ultrasound alone. However, the initial diagnosis was strongly guided by sonographic findings.

This case illustrates that ultrasound can detect paraspinal abscesses adjacent to the lumbar facet joints and may provide an important early diagnostic clue in patients presenting with persistent back pain and inflammatory signs. Adequate ultrasound equipment and operator experience are essential for reliable detection. MRI remains mandatory to confirm the diagnosis and reliably assess epidural involvement.

## Data Availability

The data supporting the findings of this case report are included within the article. Additional de-identified clinical information is available from the corresponding author upon reasonable request, subject to institutional and ethical requirements.

## Generative artificial intelligence use

The authors confirm that no generative artificial intelligence was used in the preparation of this manuscript.

